# Transcriptome and Literature Mining Highlight the Differential Expression of ERLIN1 in Immune Cells during Sepsis

**DOI:** 10.3390/biology10080755

**Published:** 2021-08-05

**Authors:** Susie S. Y. Huang, Mohammed Toufiq, Luis R. Saraiva, Nicholas Van Panhuys, Damien Chaussabel, Mathieu Garand

**Affiliations:** 1 Research Department, Sidra Medicine, Doha 26999, Qatar; mtoufiq@sidra.org (M.T.); lsaraiva@sidra.org (L.R.S.); nvanpanhuys@sidra.org (N.V.P.); dchaussabel@sidra.org (D.C.); 2 College of Health and Life Sciences, Hamad Bin Khalifa University, Doha 34110, Qatar

**Keywords:** cholesterol biosynthesis, immunometabolism, leukocytes, calcium channel, bacteremia, sepsis, neutrophil, myeloid cells, innate immunity

## Abstract

**Simple Summary:**

Sepsis is a disease affecting 19 million people worldwide, and accounts for 5 million deaths annually. Efforts in finding predictive markers of sepsis development have been difficult due to the complex clinical features of the disease. Public data repositories are valuable resources for mining gene expression changes across different studies. Using such resources, we observed a consistent increase in *ERLIN1*—a gene coding for an ER membrane prohibitin and regulator of cholesterol—in whole blood, and across a variety of immune cells, during sepsis or sepsis-like conditions. We verified this finding by exposing the whole blood of healthy volunteers to a combination of lipopolysaccharide and peptidoglycan in order to simulate sepsis. We observed an increase in ERLIN1 in whole-blood neutrophils and HL60 cell lines during sepsis; however, the protein was expressed differently in other immune blood cells. The current available studies on ERLIN1 and sepsis indicate a knowledge gap between the functions of ERLIN1, calcium balance, and cholesterol and fatty acid synthesis, and sepsis. Together with experimental data, we think that ERLIN1 is modulated differently in immune cells in response to infection, and has important implications for ER functions and/or ER membrane protein components during sepsis.

**Abstract:**

Sepsis results from the dysregulation of the host immune system. This highly variable disease affects 19 million people globally, and accounts for 5 million deaths annually. In transcriptomic datasets curated from public repositories, we observed a consistent upregulation (3.26–5.29 fold) of *ERLIN1*—a gene coding for an ER membrane prohibitin and a regulator of inositol 1, 4, 5-trisphosphate receptors and sterol regulatory element-binding proteins—under septic conditions in healthy neutrophils, monocytes, and whole blood. In vitro expression of the ERLIN1 gene and proteins was measured by stimulating the whole blood of healthy volunteers to a combination of lipopolysaccharide and peptidoglycan. Septic stimulation induced a significant increase in *ERLIN1* expression; however, ERLIN1 was differentially expressed among the immune blood cell subsets. ERLIN1 was uniquely increased in whole blood neutrophils, and confirmed in the differentiated HL60 cell line. The scarcity of ERLIN1 in sepsis literature indicates a knowledge gap between the functions of ERLIN1, calcium homeostasis, and cholesterol and fatty acid biosynthesis, and sepsis. In combination with experimental data, we bring forth the hypothesis that ERLIN1 is variably modulated among immune cells in response to cellular perturbations, and has implications for ER functions and/or ER membrane protein components during sepsis.

## 1. Introduction

Sepsis affects 19 million patients worldwide, with a mortality rate between 25% and 30%, rising to 50% when shock is present [1]. Compared with healthy adults, neonates and young infants, as well as the elderly, are more susceptible to sepsis [2,3]. Immunocompromised individuals are also at higher risk. Although palliative care and antimicrobial treatment have improved sepsis management, the mortality rate remains high due to disease heterogeneity, highly variable host characteristics (e.g., cardiovascular, immunological, etc.), and shortcomings in early detection and diagnosis [4,5].

Sepsis develops as a result of the dysregulation of innate immune responses, leading to a systemic infection [6,7]. The ensuing cytokine storm can have severe pathological effects on multiple organs, with subsequent activation of the modulatory pathways potentially causing immunoparalysis. Immunoparalysis in sepsis patients has been associated with defects in cellular and tissue metabolism that have been correlated with adverse outcomes [2,3,4]. Whilst few studies have proposed biomarker signatures for identifying patients at risk of developing sepsis prior to the manifestation of symptoms [8,9], others have identified genetic variations that increase the risk of infection or immune dysregulation, leading to the identification of several “primary immune deficiency” diseases [10,11].

The growing repertoire of publicly accessible transcriptomic datasets, such as those on the Gene Expression Omnibus (GEO), serves as a valuable resource for discovering novel predictors, diagnostic markers, and disease progression markers. Diverse collections of omics-based experiments have the potential to help elucidate the immunology and genetics underlying the heterogeneity of the pathogenesis of sepsis [12,13]. In that regard, reductionist approaches have been used to successfully identify putatively novel genes/functions [14,15,16,17] and perform system-level reanalysis [18,19,20].

Neutrophils constitute a primary type of leukocyte in peripheral blood, and play key roles in the control of infections. The crucial role of neutrophils in infection, inflammation, and immunity has been extensively reviewed by others [21,22,23,24,25,26]. Our starting point was the selection of a candidate gene from a list of differentially expressed genes (DEGs) identified in dataset GSE49755, in which neutrophils from healthy donors were exposed to septic plasma compared to healthy plasma [27]. The list of DEGs potentially contains important modulators of neutrophil activation and/or function, and is of interest to further our understanding of the pathogenesis of sepsis. A candidate gene was selected based on the following basis: (1) being upregulated in neutrophils exposed in vitro to sera of patients with sepsis and, importantly, (2) the absence of overlap between the candidate gene literature and sepsis, inflammation, or neutrophil literature, indicating that the data being examined are novel.

We identified ER lipid raft associated 1 (ERLIN1) as a suitable candidate gene, and sought to corroborate the initial finding in multiple other public transcriptome datasets in which ERLIN1 transcript levels were measured. We found that ERLIN1 gene expression was upregulated in blood cells under multiple septic conditions. Based on literature mining, the major functions of ERLIN1 are ER-associated protein degradation [28] and regulation of cholesterol synthesis [29]. Additionally, ERLIN1 has recently been identified as a significant connecting bridge between immunity and metabolism [30]. We replicated the induced expression of *ERLIN1* observed during sepsis by using in vitro models exposed to a combination of lipopolysaccharide (LPS) and peptidoglycan (PGN). From multiparameter flow cytometry, we observed contrasting cellular phenotypes of ERLIN1 abundance in leucocyte populations from cultured whole blood. Additionally, we analyzed the ERLIN1 abundance levels in the neutrophilic cell line HL60 upon LPS/PGN stimulation, and observed a differential intracellular cholesterol dynamic over time. Lastly, we discuss these findings, and how they contribute to our understanding of the pathogenesis of sepsis.

## 2. Materials and Methods

### 2.1. GEO Dataset Exploration and Analyses

The initial observation originated from the neutrophil dataset GSE49755. Briefly, isolated neutrophils from healthy donors (*n* = 2) were cultured for 6 h in the presence of sera obtained from other healthy individuals (*n* = 6) or individuals hospitalized with bacterial sepsis (*n* = 6) [27]. Two control conditions were also included: culture without serum, with or without LPS. Transcriptional profiles were generated using Illumina BeadArrays. The levels of expression and variance between sera from the control and septic groups were assessed via t-test (*p* < 0.01) and F-test (*p* < 0.01), respectively. *ERLIN1* was selected from among a set of genes whose abundance was increased in healthy neutrophils following exposure to septic sera. Additional transcriptomic datasets relevant to neutrophils and/or sepsis were retrieved from the GEO, and the associated SOFT files were uploaded to a custom data-browsing application that we recently developed—*SysInflam HuDB* [13,31]. GSE IDs mentioned in this manuscript are linked to the *SysInflam HuDB* gene expression browser (GXB); specific gene expression data can be visualized by inputting the official gene symbol in the upper-left corner of the page.

### 2.2. Literature Search and Synthesis

Literature pertaining to ERLIN1 was retrieved using a PubMed query comprised of its official symbol, name, and known aliases: (“ERLIN1[All Fields] OR Erlin-1[All Fields] OR KE04[All Fields] OR SPFH1[All Fields]”). As of 18 February 2021, this query returned 45 results, with 32 specific to humans; note that the resulting numbers of articles described in this paragraph each contain direct web links to the PubMed query result page. No overlap was found with the sepsis literature in humans (143,948 PubMed entries): (“sepsis”[MeSH Terms] OR “sepsis”[All Fields] AND “humans”[MeSH Terms]). Extending the search to literature on “Inflammation” or “Neutrophils” (497,786 and 84,188 PubMed entries, respectively) returned 2 (https://pubmed.ncbi.nlm.nih.gov/23477746/ accessed on 18 February 2013) and 0 (https://pubmed.ncbi.nlm.nih.gov/27045805/ accessed on 5 April 2016) articles overlapping with ERLIN1 literature, respectively. To obtain information about the biological functions of the ERLIN protein, key processes/terms from the titles of the 45 retrieved articles were extracted and manually assembled into 20 representative concepts. Briefly, terms such as membrane, transmembrane, lipid, lipid raft, and hydrophobic helices were combined, and the search query for those terms took the following structure: (“membrane” [MeSH Terms] OR “membrane” [All Fields] OR “transmembrane”[MeSH Terms] OR “transmembrane” [All Fields] OR “lipid” [MeSH Terms] OR “lipid” [All Fields] OR “lipid raft” [MeSH Terms] OR “lipid raft” [All Fields] OR “hydrophobic helices” [MeSH Terms] OR “hydrophobic helices” [All Fields]). Each concept was then combined with ERLIN1 and its known aliases and subjected to subsequent PubMed queries. To reduce the complexity and repetitiveness, a final list of 5 key biological concepts was synthesized from the 20 concepts; as a criterion, PubMed queries of key biological concepts combined with ERLIN1 had to result in at least three articles in order to be included in the final list. This final list of concepts was then combined with either “sepsis”, “inflammation”, or “neutrophil” and used in a series of the final PubMed queries.

### 2.3. In Vitro Stimulation Assay

Heparinized diluted whole blood (WB) samples from healthy volunteer donors were exposed to media (control) or combined LPS/PGS at 100 ng/mL and 5 ug/mL, respectively, to simulate septic conditions. Samples were collected at 6 h post-stimulation for total RNA analysis and overnight for protein expression and localization analyses. Briefly, whole blood (maximum total of 4 mL) from each donor was collected via venipuncture into a heparin-sulfate vacutainer (Becton Dickinson (BD), Franklin Lakes, NJ, USA) and mixed at a 1:1 ratio with RPMI (Thermo Fisher, Waltham, MA, USA). Next, 500 μL of WB:RPMI mixture was added to cryovials or microwells already containing the specific stimulation. The samples were incubated in a tissue culture incubator at 37 °C with 5% CO_2_ for the desired time. For transcript abundance, the 6-h incubation was optimally selected based on previously reported time-course data for *ERLIN1* expression (GSE3284) [32]. The experiment was repeated twice.

### 2.4. HL60 Culture, Differentiation, and In Vitro Stimulation

HL60 cells (ATCC, Manassas, VA, U.S., cat#CCL-240) were cultured in Nunc T25 flasks in Iscove’s Modified Dulbecco’s Medium (ATCC) supplemented with 20% fetal bovine serum (HyClone). For differentiation, cells were further supplemented with 1.3% *v*/*v* dimethyl sulfoxide for six days prior to the in vitro stimulation assay. Viability and cell count were monitored using Via1-Cassette and NucleoCounter NC-200TM (ChemoMetec, Allerod, Denmark). For the stimulation assay, one million cells were added to the wells of a 96-well u-bottom culture plate. The final concentrations of LPS and PGN in the wells were 100 ng/mL and 5 ug/mL, respectively. The experiment was repeated three times.

### 2.5. ERLIN1 Knock-Out in HL60 Cell Line

We designed four guide RNA (gRNA) sequences (Integrated DNA Technologies, Coralville, IA, U.S.) to target exon 6 of *ERLIN1*, as this exon is common across the existing alternative spliced transcript variants recorded in the NCBI database. The number of possible off-target coding sequences ranged between 9 and 13, with at least 2 mismatches. The Doench score for the gRNA ranged between 0.510 and 0.762, and the appropriate size of the in vitro transcribed product was verified by gel electrophoresis. Electroporation of 4 × 10^5^ HL60 cells was performed with the Neon Transfection System (Thermo Fisher) using a Cas9:gRNA ratio of 1.5 ug:375 ng. Next, 72 h after gRNA transfection, DNA was extracted, and PCR was performed in preparation for Sanger sequencing. PCR quality and quantity were assessed by gel electrophoresis. The CRISPR-Cas9-mediated cleavage efficiency was estimated with the GeneArt Genomic Cleavage Detection Kit (Thermo Fisher). Tracking of indels by decomposition (TIDE) was used to estimate the CRISPR/Cas9 modification efficiency.

For cloning and subsequent amplification, single-cell sorting of HL60 cells transfected with ERLIN1 gRNA2 (C2) and gRNA4 (C1) was performed with 50% conditioned media and 10-uM ROCK inhibitor (Sigma-Aldrich). Forward scatter and side scatter with doublet discrimination and autofluorescence exclusion were applied during sorting. Clones were cultured for three weeks in 96-well plates at 37 °C with 5% CO_2_. Ten clones were randomly selected to verify the stability and sustainability of the gene edition. Each clone was then analyzed individually by PCR (exon 6 region) and TIDE analysis. Five clones were validated, and all mutations reported were verified and resulted in an early stop codon and disruption of the protein sequence.

### 2.6. RNA Extraction and qPCR

At the time of collection, cultured whole blood and HL60 samples were mixed with PAXgene™ reagent (Qiagen, Venlo, Netherlands) at a sample-to-reagent ratio of 1:2.76, and then gently inverted and stored at −80 °C within two hours. Total RNA extraction was performed with the PAXgene™ Blood RNA Kit (Qiagen) according to the manufacturer’s protocol. cDNA was synthesized from 500 ng of total RNA using the SuperScript™ III First-Strand Synthesis System (Thermo Fisher) and analyzed by qPCR using SYBR Green PCR Master Mix (Thermo Fisher, USA) on a QuantStudio system (Thermo Fisher, USA) with the following thermal cycles: initial denaturation at 95 °C for 10 min, 40 cycles of denaturation at 95 °C for 15 s, and annealing/extension at 60 °C for 1 min. For melting curve analysis, the following thermal parameters were used: (1) 95 °C for 15 s, (2) 60 °C for 1 min, (3) 95 °C for 15 s, and (4) 4 °C, maintained. Transcripts of interest (RefSeq accession number in parentheses) were detected using the following primer pairs (F: forward, R: reverse): *ERLIN1*-1 (NM_001100626.1)-F 5′-GAAAGCTCACTCCCCTCTAAG-3′, R 5′ TGTTCCCACTTAACCCCTTG-3′: *GAPDH* (NM_002046.7)-F 5′-GAAGGTGAAGGTCGGAGTC 3′, R 5′-GAAGATGGTGATGGGATTTC-3′. Target gene expressions were normalized to GAPDH expression, and are shown relative to the control samples (ΔΔCt method).

### 2.7. Flow Cytometry

Cultured whole blood and HL60 phenotyping and immunostaining for surface and intracellular markers were performed using the standardized protocol published by Cytobank [33]. Briefly, cells previously frozen in FACS lysing solution (BD, USA) were thawed for 15 min in a 37 °C water bath and washed twice in stain buffer (BD). Samples were then permeabilized with Phosflow Perm Buffer II (BD), washed twice in stain buffer, incubated with FC block (cat# 564220, BD) for 10 min at room temperature, and stained by adding normal goat serum (10 uL, cat#0060-01, Southern Biotech, Birmingham, AL, USA) and the recommended volume of antibodies; fluorescence-minus-one controls were also prepared for each fluorochrome. After a 60-min incubation at room temperature in the dark, cells were washed twice, and incubated for 45 min with goat anti-rabbit Alexa488 (1/5000) secondary antibody. During incubation, compensation controls were prepared using UltraComp eBeads (Invitrogen). Cells were then fixed in 4% PFA (Invitrogen), washed twice, and resuspended in stain buffer prior to flow cytometric analysis. The fluorochrome-labelled antibodies used were CD45 V500 (HI30, RRID:AB_1937324, BD), HLA-DR Pacific Blue (L243, RRID: AB_2561913, BioLegend, San Diego, CA, USA), rabbit anti-human ERLIN1 primary (Human Protein Atlas number: HPA011252, Sigma-Aldrich, Burlington, MA, U.S.), goat anti-rabbit superclonal secondary antibody Alexa 488 (RRID:AB_2536097, Thermo Fisher), Fixable Viability Dye (FVD) UV495 (cat# 423107, BioLegend), CD11b BUV661 (D12, RRID:AB_2874279 BD), CD16 PE (3G8, RRID:AB_2563801, BioLegend), CD11c PE-Dazzle594 (3.9, RRID:AB_314176, BioLegend), CD3 PE-Cy7 (UCHT1, RRID:AB_2738196 BD), and CD66b APC-Cy7 (G10F5, RRID:AB_2750184, BioLegend). Acquisition was performed on a BD FACSymphony A5 (BD). Compensation beads were used to standardize the voltage settings, and used as the single-stain positive and negative controls. A minimum of 100,000 uncompensated events were acquired from each sample, and compensation was set in FlowJo V.10 (FlowJo, Ashland, OR, USA). Gating during analysis (Figure S1) was based on the fluorescence-minus-one principle [34,35]. Cell viability was assessed via forward and side scatter (FSC/SSC) appearance, as previously described [36,37], or with the Zombie UV Fixable Viability Kit (BioLegend). The minimum number of gated cell events accepted for analysis was 100 events. The experiments were performed in duplicate and repeated twice.

### 2.8. Intracellular Cholesterol Levels

Total intracellular cholesterol content from cultured whole blood and cell line samples was quantified using Amplex Red Cholesterol Assay (Thermo Fisher) as per the manufacturer’s protocol. Briefly, samples were homogenized in lysis buffer containing 50 mM Tris-HCl pH 7.5, 150 mM NaCl, 1% Nonidet P-40, 0.5% desoxicolate, and protease inhibitors diluted in reaction buffer. Samples were then sonicated for 4 cycles of 1 min with 30-s intervals on ice. After sonication, an equivalent volume of Amplex Red working solution (300 μM Amplex Red, 2 U/mL cholesterol oxidase, 2 U/mL cholesterol esterase, and 2 U/mL horseradish peroxidase) was added to each sample. The samples were then incubated at 37 °C for 30 min in the dark and the absorbance measured at 535 nm using a spectrophotometer (Microplate Reader Model 550, Bio-Rad, Hercules, CA, USA). Quantification of total cellular protein, for normalization of cholesterol concentrations, was performed using the Pierce BCA Protein Assay Kit (Thermo Fisher) according to the manufacturer instructions. 

### 2.9. Statistical Analyses

GraphPad Prism v.5 (GraphPad, San Diego, CA, U.S.) was used for all plots and statistical analyses. Outliers were determined by the removal of outliers (ROUT) method (Q = 1%) and removed from median calculation. For any two-group comparisons, a two-tailed unpaired t-test (*p* < 0.05) was used to identify significant differences. For comparison of three groups or more, the Brown–Forsythe test and Welch’s one-way ANOVA were used, and multiple *p*-values were determined by unpaired t-test with Welch’s correction. Two-factor ANOVA, followed by Tukey’s test, was used to test for the significant effects of time and stimulation on total intracellular cholesterol levels in HL60 cell lines.

## 3. Results

Public repositories of articles and data—such as PubMed and the GEO—represent vast resources, but can be complicated to explore. We implemented a logical reductionist approach to investigate putative novel biomarkers in sepsis; however, this could be applied to any field of research.

The steps consist of (1) identifying a gene of interest based on its differential expression in the pathological/physiological context of interest, (2) validating the reproducibility of the initial observation, (3) determining the current body of literature linking the gene and topic, (4) extracting the known biological concepts regarding the gene, and (5) inferring putative novel roles to the gene, with support from literature.

### 3.1. Experimental Validations

We initially observed a significant induction of *ERLIN1* expression (5.29-fold; *p* < 0.01) in healthy neutrophils exposed to septic plasma compared with healthy plasma controls (GSE49755, see Materials and Methods). Moreover, similar increases in ERLIN1 expression in sepsis were also observed in other transcriptome datasets obtained from whole blood (*n* = 8) and isolated cell (*n* = 8) samples, and generated from in vivo (*n* = 5), ex vivo (*n* = 1), and in vitro (*n* = 8) experimental designs (Table 1). The induction of ERLIN1 was seen in both adult and neonate septic cohorts infected by multiple pathogens. These findings are consistent with the transcriptomic changes reported in various human immune cells under septic conditions (GSE60424) [38].

The etiology of sepsis can often be polymicrobial, with the detection of both Gram-positive and -negative bacteria [39,40,41,42]. Thus, to validate the aforementioned observations, we collected whole blood from healthy individuals and cultured it for six hours with a combination of LPS and PGN. As the full extent of the immune response likely involved independent as well as synergistic effects [43], we employed both TLR4 and TLR2 to stimulate immune cells from the blood and to artificially recreate a multiorganism infection. The ligand concentrations used are known to generate reliable innate immune responses [36,44]. The experimental design was to create an additive effect and maximize the “severity” of the immune stimulation, with polymicrobial sepsis being associated with higher risk for complications and mortality [41].

**Figure 1:**
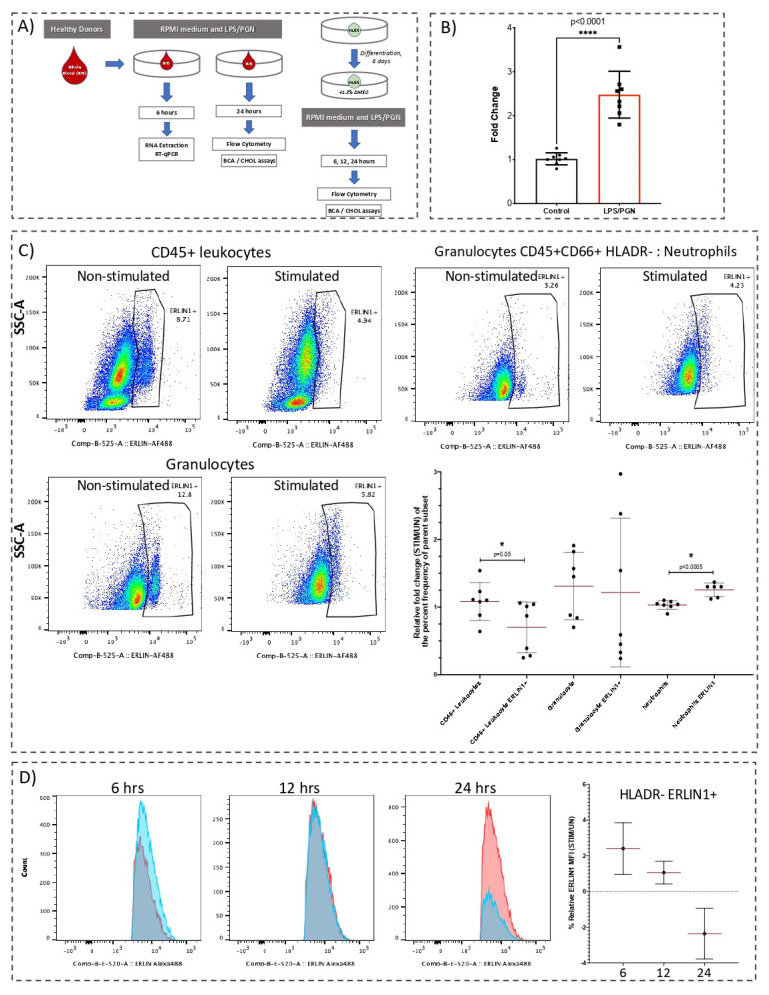
Abundance of ERLIN1 transcript and proteins in cultured whole blood and HL60 cells in response to LPS/PGN exposure. (**A**) Experimental designs for in vitro stimulation of cultured whole blood and HL60 cells. Whole blood was incubated with or without (UN) a combination of lipopolysaccharide (LPS, 100 ng/mL) and peptidoglycan (PGN, 5 ug/mL) for 6 h with stimulation prior to total RNA extraction and RT-qPCR quantification. Determination of protein levels by flow cytometry and cholesterol assays was performed after 24-h stimulation. For HL60, cells seeded at 3E06 cells/mL were differentiated for six days prior to stimulation for 6, 12, and 24 h with LPS/PGN, followed by processing for flow cytometry and cholesterol assays as described in the Materials and Methods section. (**B**) Whole blood from healthy donors (*n* = 8) after 6 h with LPS/PGN or culture medium (control). *ERLIN1* expression was assessed by RT-qPCR and normalized to GAPDH transcript expression. (**C**) Representative dot plots of ERLIN1 abundance in cultured whole blood immune cells before and after stimulation for 24 h (expressed as % of parent cell population). After culture, cells were harvested in BD FACS Lyse and stored at −80 °C until staining. Immunostaining and flow cytometry were performed as described in the Materials and Methods section. Gates were calibrated using all-minus-one staining mix (Figure S4). The summary of the main findings shows the relative fold change (STIM/UN) of the percentage frequency of the parent subset. (**D**) Relative abundance of ERLIN1 in HL60 cells at 6, 12, and 24 h, expressed as the fold change in median fluorescence intensity (MFI) for each stimulation time (stimulated/non-stimulated); blue and red histograms represent stimulated and non-stimulated, respectively. * *p*-value ≤ 0.05. **** *p*-value < 0.0001.

In line with the previous observations, blood exposed to LPS/PGN displays a significant increase (2.6-fold; *p* < 0.01) in ERLIN1 gene expression, when compared with control conditions (*n* = 8, Figure 1A,B). Notably, the magnitude of induction of *ERLIN1* in whole blood was less than in purified neutrophils, which may be indicative of the differences in milieu and/or the interdependence with other cell subsets. Immune responses are known to show intraday variance, which constitutes a limitation in our study; nevertheless, the increase in *ERLIN1* expression was repeatably observed in our experiment, and across multiple public datasets.

ERLIN1 abundance in cultured whole blood was determined by flow cytometry. The frequency of ERLIN1+ whole blood neutrophils was significantly increased by 24-h LPS/PGN stimulation, while the ERLIN1+ frequency in leukocytes was significantly reduced (*n* = 7, Figure 1C). In the granulocyte subset, we observed a decreasing trend in ERLIN1 abundance, but with high interindividual variance. The intrinsic levels of ERLIN1 in other identifiable cell subsets were highly variable and/or seemingly affected by the abundance of the parent population; thus, those cell subsets were not included in further analyses. In contrast, the relatively low variance and high consistency of ERLIN1 abundance in the neutrophil subset is noteworthy. To explore this avenue further, we used the neutrophilic cell line HL60. Stimulation with LPS/PGN for 6 h resulted in a modest increase in the relative frequency (stimulated/non-stimulated) of ERLIN1+ HL60 cells, while no distinguishable changes were observed at 12 and 24 h (*n* = 3 to 4, Figure S2). However, the relative changes (stimulated/non-stimulated) in the abundance (median fluorescence intensity, or MFI) of ERLIN1 decreased over time (2.4, 1.06, and −2.36 fold change, at 6, 12, and 24 h, respectively; n = 3; Figure 1D).

### 3.2. Literature Evaluation between ERLIN1 and Sepsis

**Figure 2:**
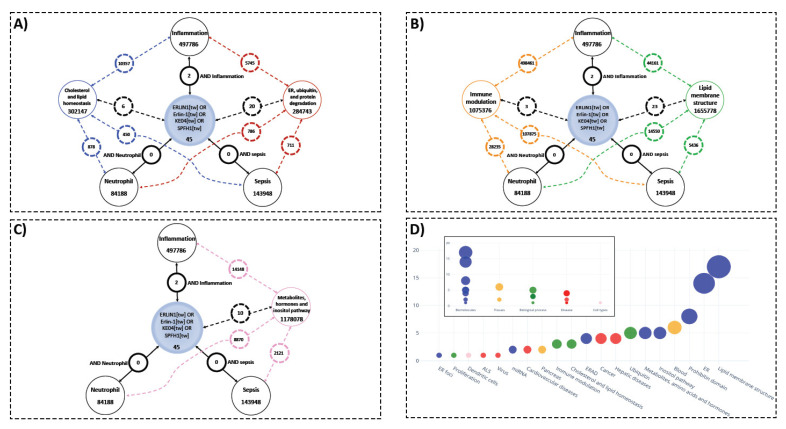
Knowledge gaps concerning ERLIN1, key biological concepts, and PubMed literature on sepsis, inflammation, and neutrophils demonstrate potential novel roles of ERLIN1 in the pathogenesis of sepsis. (**A**–**C**) The available literature used to infer potential roles for ERLIN1 expression in immune cells during sepsis through ER-mediated protein degradation, cholesterol homeostasis, and signaling events via lipid structures and inositol pathways. Results of literature search for *ERLIN1* (and aliases) and the terms sepsis, inflammation, and neutrophils in humans are enclosed in black circles; the numbers represent the article count retrieved for each association. The PubMed search results with the key biological concepts (colored circles) and literature on sepsis, inflammation, and neutrophils are depicted with dotted lines of corresponding colors. (**D**) Functional grouping of the title terms from the PubMed query on ERLIN1. The extracted terms were manually assembled into 20 representative functional groups (main graph), and then further condensed to a final list of five key biological concepts for subsequent search strategies.

To assess the current literature’s understanding of the role of ERLIN1 in the context of sepsis, we performed PubMed searches on ERLIN1, which returned 45 articles specific to humans, while no literature to date has linked this molecule to sepsis or neutrophils, and with only 2 articles having mentioned inflammation (Figure 2A). A general review and identification of the main ERLIN1 research themes was achieved by manually extracting the biological concepts from ERLIN1 literature (see Materials and Methods). Although there was no direct overlap between the ERLIN1 and sepsis literature, the two may be linked by intermediate biological concepts, such as those linking ERLIN1 with inflammatory processes. Thus, the described biological functions of ERLIN1 suggest potential roles for its expression during sepsis in ER-mediated protein degradation, cholesterol homeostasis, and signaling events through lipid membrane structures and inositol pathways (Figure 2B–D).

## 4. Discussions

**Figure 3:**
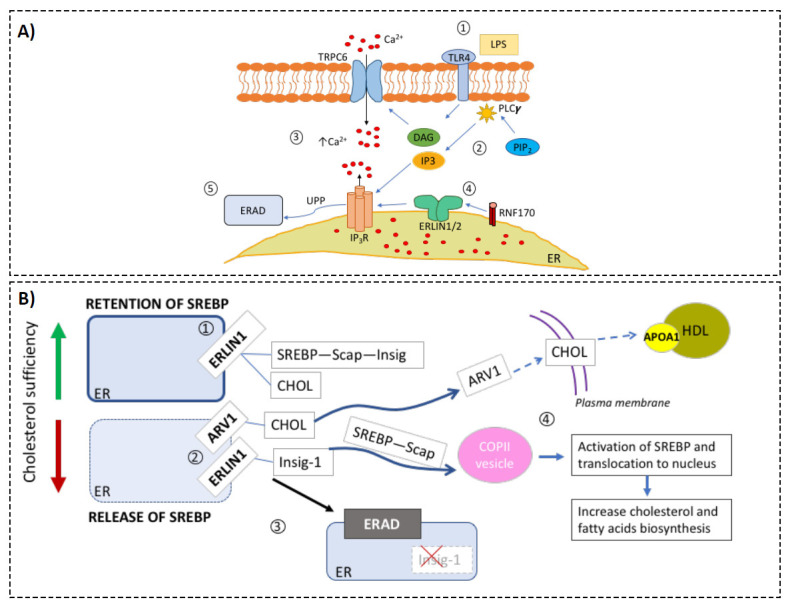
Hypothetical model of the roles of ERLIN1 in cellular metabolic regulation during sepsis. Based on the compendium of evidence from public transcriptomic data, scientific literature reports, and our preliminary results, we assembled relevant biological knowledge about the roles of ERLIN1 in calcium and cholesterol regulation. (**Panel A**) ERLIN1-mediated regulation of intracellular Ca^2+^ during sepsis: (1) Toll-like receptor 4 (TLR4) is activated by its ligand l LPS; (2) Second messengers, inositol triphosphate (IP3), and diacylglycerol (DAG) are produced via the enzymatic activity of phospholipase-c (PLCγ); (3) increase in intracellular Ca^2+^ due to activation of the transient receptor potential ion channel TRPC6 by DAG, and release of ER pool via the binding of IP3 to the inositol triphosphate receptor (IP_3_R); (4) immediately upon IP3R activation, ERLIN dimer recruits E3 ligase RNF170 [9], and (5) tags the receptor for degradation via ERAD. (**Panel B**) Putative alteration of cholesterol homeostasis during sepsis: (1) ERLIN1 retains cholesterol (CHOL) and the SREBP–SCAP–Insig complex in the ER membrane [7]; (2) SCAP-induced conformation changes during CHOL insufficiency and leads to dissociation of SREBP from ERLIN1. Independently, CHOL is transported to the plasma membrane via the sterol homeostasis protein (ARV1); (3) ERLIN1 tags Insig-1 for degradation, and the SREBP–SCAP complex is released to the Golgi to activate CHOL synthesis. Septic stress could induce ERLIN1 expression to increase ERAD efficiency. Collaterally, this could promote the retention of intracellular cholesterol. Low levels (below 25.1 mg/dL) of high-density lipoprotein cholesterol (HDL-C) have been strongly associated with the risk of adverse outcomes in sepsis [8].

ERLINs were first characterized from the cholesterol-enriched, detergent-resistant membrane fractions in human myelomonocytic cells, and contain conserved domains of prohibitins [45]. Uniquely to the prohibitin family, ERLINS are localized to the ER [46], in the lipid-raft-like domains. The best defined function of the ERLIN1/2 protein complex is to mediate the ER-associated protein degradation of inositol 1, 4, 5-trisphosphate receptors (IP3Rs) [28,47,48]. IP3Rs are pivotal in a plethora of signaling pathways dependent on cytoplasmic Ca^2+^ [49]. Figure 3A depicts the known physiological roles of ERLINs regarding calcium homeostasis. In brief, ERLINs mediate IP3R degradation via E3 ubiquitin-protein ligase RNF170 recruitment [50]. RNF170 mediates the ubiquitination and processing of IP3R via the ERAD pathway. *RNF170* expression was upregulated in whole blood during sepsis (e.g., GSE57065, GSE63311; Figure S3A,B), particularly in neutrophils and macrophages, and has been suggested to affect TLR3 ubiquitination/degradation [51]. Additionally, induction of *RNF170* was more profound in sepsis compared with systemic inflammatory response syndrome (e.g., GSE9960; Figure S3C), suggesting distinct signaling events at the level of calcium metabolism and degradation of IP3R. Another catabolic process, autophagy, has also been shown to be mediated by autophagy and beclin 1 regulator 1 (AMBRA1) and ERLIN1 complexes within the mitochondria-associated membranes [52].

Calcium homeostasis likely plays an important role in the pathogenesis of sepsis. Animal models have shown that sepsis-induced cardiomyopathy develops as the result of myocardial Ca^2+^ dysregulation (reviewed in [53]). Endotoxicosis and sepsis altered IP3-dependent Ca^2+^ release in rat hepatocytes, which partly accounted for the metabolic and functional changes associated with these pathologic states [54]. In humans, these two conditions were modulated by calcium channel blockers (reviewed in [55]). In human cholangiocytes, LPS decreased type-3 IP3R mRNA and protein levels [56]. Additionally, we found that *ITPR1* expression (the gene coding for IP3R) was decreased during sepsis in GSE57065 and GSE29536 (Figure S3D).

Under stressful conditions, the functional homeostasis of the ER is perturbed [57]. ER stress is pivotal in regulating cell signaling—particularly that of the immune response [58,59]. Additional evidence supports the importance of dysregulation of ER function, and/or its membrane protein component, in the pathogenesis of sepsis [60,61,62]. Following cecal ligation, septic mice exhibited higher ER stress compared with non-septic mice, with increased accumulation of unfolded proteins and activation of proteolysis [63]. As such, *CHOP* (*DDIT3;* *GADD153*; Gene ID 1649)—an indicator of ER stress [64]—was increased during sepsis (GSE57065 and GSE29536; Figure S3E). In addition to IP3Rs, ERLINs are involved in the endoplasmic-reticulum-associated degradation (ERAD) of several proteins [65,66]. As the ERLIN complex is known to affect the stability of some model ERAD substrates [28], increased ERLIN1 during the acute phase of sepsis may triage proteins for secretion or degradation [67]. Thus, ERLIN1 could indirectly participate in the regulation of immune signaling by modulating the recognition of degradation targets by ERAD [68,69,70].

ERLINs are also important for the regulation of cholesterol synthesis [29] (Figure 3B). The presumed bridge between sepsis and ERLIN1 is supported by the characteristically low levels of high-density lipoprotein cholesterol (HDL-C) and triglycerides observed in the plasma of septic patients, which are strongly associated with risk of adverse outcome [71,72,73,74,75,76]. ERLINs are regulators of sterol regulatory element-binding proteins (SREBPs—key transcription factors for cholesterol and fatty acid biosynthetic genes [29,77]. A recent trans-disease meta-analysis revealed four co-regulated loci between psoriasis and type 2 diabetes, one of which corresponded to the ERLIN1 gene; the resulting proteins are connected through the NF-κB signaling pathway [30]. This finding provides support to the concept of immune cell metabolic dysregulation as a means of disease progression. In this context, a better molecular understanding of the cause of septic progression could yield early biomarker signatures which, in turn, could lead to more effective treatments [78].

Interestingly, a recent study indicated that ERLIN1 is among the major contributors to the monounsaturated-to-saturated fatty acid ratio [79]. During cholesterol deficiency, ERLIN1 is dissociated from SREBP, and tags INSIG-1 for degradation, leading to the release of the SREBP–SCAP complex to the Golgi to activate cholesterol synthesis. It should be noted that both *SCAP* and *INSIG-1* expression in neutrophils remained unchanged with exposure to septic plasma (GSE49755; Figure S3F,G). Our preliminary data showed that LPS/PGN stimulation did not change intracellular cholesterol concentration in cultured whole blood; it is possible the variable abundance/modulation of ERLIN1 among blood cells (Figure 1C) masks the changes in specific cell subsets. However, in differentiated HL60 with neutrophil-like phenotypes, total intracellular cholesterol was significantly increased at 6 h post-stimulation but reduced by 12 and 24 h (Figure S4**)**. This trend mirrored the abundance of ERLIN1 determined by flow cytometry (Figure 1D). The changes in cholesterol were absent, however, after ERLIN1 knockout (Figure S5). Thus, ERLIN1 may play an important role in the homeostasis between proteolysis needs, membrane fluidity, and cholesterol, which is critical for normal cellular function.

Plasma membrane cholesterol content improves raft composition, and possibly TLR function [80]. SREBPs increase the biosynthesis of fatty acids and induce the production of IL-1β via NLRP3/inflammasome activation in macrophages [81]. Interestingly, in GSE49755, *SREBF1* was downregulated in septic neutrophils (Figure S3H), suggesting a diminution of its own positive feedback loop [82]. IL-1β expression was also decreased during sepsis (GSE49755, Figure S3I). Furthermore, LPS stimulation downregulated apolipoprotein A1 (APOA1) expression, which is involved in the efflux of cholesterol from the cells, suggesting that sepsis can lead to increased cellular cholesterol levels [83].

Non-septic stress can also induce *ERLIN1* expression, as seen in monocytes from healthy donors, patients with metastatic breast cancer or tuberculosis (GSE65517; Figure S3J). While the role of ERLIN1 in cancer and tuberculosis is undefined, these observations suggest that ERLIN1 may be an indicator of the disrupted intracellular environment in disease states. *ERLIN1* expression was increased in neonates with necrotizing colitis or bacterial infection, but not in neonates with viral infections (GSE25504-6947, GSE25504-13667; Figure S3K,L). Recently, ERLIN1 was shown to be required for hepatitis C virus and simian virus 40 infections [62,84]. Due to its ER location and effect on membrane composition, the involvement of ERLIN1 may be dependent on the type of infection. Lastly, ERLIN1 is part of a central node of interactors with coronavirus non-structural proteins and, together with the increased *IP3R* expression during SARS-CoV-2 infection, suggests a putatively important role in coronavirus infections via the modulation of intracellular calcium signaling [85].

## 5. Conclusions

Through analysis of transcriptomic datasets present in the publicly accessible GEO database, we revealed that *ERLIN1* expression was increased during septic conditions. Calcium flux, ER stress, ubiquitin-mediated protein degradation, and lipid membrane modularity are processes and concepts known to be influenced by ERLIN1, and are indicative of the potential roles of ERLIN1 induction in the context of sepsis. Although our literature mining and experimental evidence points to the putative impact of ERLIN1 in the pathogenesis of sepsis, its roles in the interplay between intracellular Ca^2+^ release and IP3R degradation as well as cholesterol depletion during sepsis remain a matter for educated speculation. Our findings suggest a novel association between ERLIN1 and sepsis. We hope that our results set the scene for future investigative studies of a more mechanistic nature, such as to block/deplete ERLIN1 and reassess intracellular cholesterol, inflammasome activation, and TLR4 signaling following stimulation with synthetic ligands or septic plasma. A thorough investigation of the role of ERLIN1 may provide novel insights into the connection between intracellular Ca^2+^ and cholesterol homeostasis and the pathogenesis of sepsis.

## Figures and Tables

**Table 1:** Changes in ERLIN1 gene expression during sepsis in additional relevant datasets. The table includes the GEO ID number for each dataset (GSE), as well as the title of the dataset as recorded on the GEO database. In addition, we report for each dataset the cell types employed, the total number of samples, the conditions compared, the fold change (FC) observed with hyperlinks to the *SysInflam HuDB* page (for instant visualization), and the *p*-value calculated for the reported comparison/FC.

Datasets	Title	Cell Types	No. of Sample	Conditions Compared	FC **	*p*-Value *
In vivo						
GSE30119	Genome-wide analysis of whole blood transcriptional response to community-acquired Staphylococcus aureus infection in vivo-GSE30119	Whole Blood	143	Patients with S. aureus infection vs healthy controls	1.34	**<0.001**
GSE54514	Whole blood transcriptome of survivors and nonsurvivors of sepsis-GSE54514	Whole Blood	163	Patients admitted to the intensive care unit with sepsis (Non Survivor) vs healthy controls	1.18	**0.008**
GSE25504 (GPL570)	Whole blood mRNA expression profiling of host molecular networks in neonatal sepsis: GSE25504 (GPL570)	Whole Blood	5	Neonates with sepsis vs healthy controls	6.12	0.04
GSE25504 (GPL13667)	Whole blood mRNA expression profiling of host molecular networks in neonatal sepsis: GSE25504 (GPL1366)	Whole Blood	20	Neonates with sepsis vs healthy controls	2.49	**<0.001**
GSE25504 (GPL6947)	Whole blood mRNA expression profiling of host molecular networks in neonatal sepsis - GSE25504 (GPL6947)	Whole Blood	63	Neonates with sepsis vs healthy controls	1.35	**<0.001**
GSE13015	Genomic Transcriptional Profiling Identifies a Blood Biomarker Signature for the Diagnosis of Septicemic Melioidosis-GSE13015-Healthy-Melioidosis-Other Sepsis-T2D	Whole Blood	39	Patients with sepsis caused by B.pseudomallei vs sepsis caused by other pathogens	7.1	**<0.001**
GSE66890	Expression of Neutrophil-related genes in patients with early sepsis-induced ARDS-GSE66890	Whole Blood	62	Patients with sepsis + acute respiratory disease syndrom vs patients with sepsis alone	1.22	0.18
Ex vivo						
GSE64457	Marked alterations of neutrophil functions during sepsis-induced immunosuppression-GSE64457	Neutrophils	23	Patients with sepsis vs healthy controls	1.27	0.47
In vitro						
GSE11755	Gene expression profiling in pediatric meningococcal sepsis reveals dynamic changes in NK-cell and cytotoxic molecules-GSE11755	Lymphocytes, monocytes, Whole Blood	41	Monocytes from children with meningococcal sepsis vs monocytes from matched healthy controls (8 hrs)	2.06	**0.001**
GSE49753	A Transcriptomic Reporter Assay Employing Neutrophils to Measure Immunogenic Activity of Septic Patients’ Plasma (DC)-GSE49753	Dendritic Cells	40	Monocyte derived dendritic cells from healthy individuals exposed to plasma from patients with sepsis vs plasma from uninfected controls	1.17	0.293
GSE49754	A Transcriptomic Reporter Assay Employing Neutrophils to Measure Immunogenic Activity of Septic Patients’ Plasma (PBMC)-GSE49754	PBMC	40	PBMCs from healthy individuals exposed to plasma from patients with sepsis vs plasma from uninfected controls	1	0.97
GSE49755	A Transcriptomic Reporter Assay Employing Neutrophils to Measure Immunogenic Activity of Septic Patients Plasma GSE49755 - Neutrophil	Neutrophils	40	Polymorphonuclear neutrophils from healthy individuals exposed to plasma from patients with sepsis vs plasma from uninfected controls	5.29	**<0.001**
GSE49756	A Transcriptomic Reporter Assay Employing Neutrophils to Measure Immunogenic Activity of Septic Patients’ Plasma (Expt. 2)- GSE49756	Neutrophils	49	Polymorphonuclear neutrophils from healthy individuals exposed to plasma from patients with sepsis vs plasma from uninfected controls	5.66	**0.014**
GSE49757	A Transcriptomic Reporter Assay Employing Neutrophils to Measure Immunogenic Activity of Septic Patients’ Plasma (Expt. 3)-GSE49757	Neutrophils	56	Polymorphonuclear neutrophils from healthy individuals exposed to plasma from patients with sepsis vs plasma from uninfected controls	4.43	**0.004**
GSE16837	Gene expression data from S. aureus-exposed neutrophils-GSE16837	Neutrophils	113	Polymorphonuclear neutrophils from healthy individuals exposed to S. aureus (strain 10254) vs unexposed (3 hrs)	4.69	**<0.001**
GSE40636	PGN induced transcriptional changes in human neonatal neutrophils-GSE40636	Neutrophils	6	cord blood purified neutrophils stimulated with peptidoglycan vs unstimulated	3.82	0.024

Notes: In vivo: Experimentation or measurements done in whole, living organism or cells without alteration of natural conditions (aside from the collection method). Ex vivo: Experimentation or measurements done in or on tissue from an organism in an external environment with minimal alteration of natural conditions; e.g. purification of specific cell types. In vitro: Experimentation or measurements done in or on whole or altered tissue from an organism in an altered external environment; e.g. stimulation of cultured biological specimens. * If F-test is non-significant (*p* > 0.01), then a two-tailed t-test for equal variance was used. * If F-test is significant (*p* < 0.01), then a two-tailed t-test for unequal variance was used. * A *p*-value of < 0.01 indicates significant differential expression of ERLIN1 between groups; indicated with bold characters. ** Fold change (FC) values contain hyperlink to GXB (underlined).

## Data Availability

The data that support the findings of this study are derived from the following resources available in the public domain: NCBI Gene Expression Omnibus (GEO) at (https://www.ncbi.nlm.nih.gov/geo/ accessed on 30 July 2021), reference numbers are mentioned in the text. Our in-house-developed GXB application, SysInflam HuDB, is publicly available at http://sepsis.gxbsidra.org/dm3/geneBrowser/list. accessed on 30 July 2021).

## Supplementary Materials

The following are available online at https://www.mdpi.com/article/10.3390/biology10080755/s1. Figure S1: Gating strategy for identifying immune cell subsets. After doublets exclusion and live-dead selection, we used size and granularity scatters (SSC-A, FSC-A) to identify leukocytes and granulocytes. From granulocytes, we further gated for neutrophils based on CD45+ and CD66+ expression. From the leukocytes gate, we gated for CD45+, then excluded CD66. From this CD66- gate, we created quadrant based on HLA-DR and CD11b expression. CD11b+/- and HLA-DR- cells were further gated with CD3 and CD16, separating NK and T lymphocytes. CD11b+ and HLA-DR+ represented monocytes. CD11b- and HLA-DR+ cells were further gated based on CD11c to separate classical DC. Figure S2: Summary of the temporal expression of ERLIN1 in HL60 cells with and without stimulation assessed by flow cytometry. A) Representative expression of ERLIN1 in cultured HL60 cells before and after stimulation for 24h (expressed as % of parent cell population). Briefly, cells were differentiated with 1.3% DMSO for six days prior to in vitro stimulation. After culture, cells were harvested in BD FACSLyse and store at -80oC until staining. Immunostaining and Flow cytometry was performed as described in Methods. B) The summary of the main findings shows the relative fold change (STIM/UN) of the percent frequency of the parent subset for each time point. Figure S3. Gene expression data from the indicated GSE ID can be easily visualized on the GXB platform we previously developed as part of our curated transcriptomic dataset collection (unpublished and currently under review, Toufiq et al. 2021, SysInflam HuDB). Figure S4. Intracellular cholesterol concentration in cultured whole blood and HL60 cell line exposed to media (control) or a combination of LPS and PGS (see Methods). Total intracellular cholesterol content was quantified using Amplex Red Cholesterol Assay. Each point represents the average of three technical replicates from a blood volunteer (A) or three independent experiments with HL60 cells (B). Shown is the average and standard deviation. Figure S5. Intracellular cholesterol concentrations in ERLIN1 knock-out HL60 cell lines exposed to media (control) or a combination of LPS and PGS (see Methods). Total intracellular cholesterol content was quantified using Amplex Red Cholesterol Assay. Each point represents the average of three independent experiments with the HL60 knockout cell lines (the knockout clone ID number is indicated on each plot). Shown is the average and standard deviation.

## Abbreviations

APOA1	Apolipoprotein A1
ARV1	Sterol homeostasis protein ARV1
CHOP	C/EBP homologous protein
ERAD	Endoplasmic-reticulum-associated protein degradation
ER	Endoplasmic reticulum
ERLIN1	ER lipid raft associated 1, human protein symbol
*ERLIN1*	ER lipid raft associated 1, human gene symbol
GXB	Gene expression browser
HDL	High-density lipoprotein
Insig-1	Insulin-induced gene 1
LPS	Lipopolysaccharide
PGN	Peptidoglycan
SCAP	SREBP cleavage-activating protein
SREBP	Sterol regulatory element-binding protein
SysInflam HuDB	Customized GXB platform, (http://sepsis.gxbsidra.org/dm3/landing.gsp, accessed on 30 July 2021)
WB	Whole blood
