# Supplementary material for: Transcriptome and Literature Mining Highlight the Differential Expression of ERLIN1 in Immune Cells during Sepsis
Source: Biology (Basel). 2021 Aug 5;10(8):755. doi: 10.3390/biology10080755 (PMC8389572; doi:10.3390/biology10080755)
Supplement: Supplementary file 1 [file biology-10-00755-s001.zip › biology-1308344-supplementary.pdf]

Transcriptome and literature mining highlight the differential expression of ERLIN1 in immune cells during sepsis.

Susie S.Y. Huang*[1], Mohammed Toufiq[1], Luis R. Saraiva[1,2], Nicholas Van Panhuys[1], Damien Chaussabel[1], Mathieu Garand*[1]

[1] Sidra Medicine, Doha, Qatar.
[2] College of Health and Life Sciences, Hamad Bin Khalifa University, Doha, Qatar

* Corresponding authors: susie.sy.huang@gmail.com (SSYH) and mathieu.garand@gmail.com (MG)

Supplementary Information (SI)

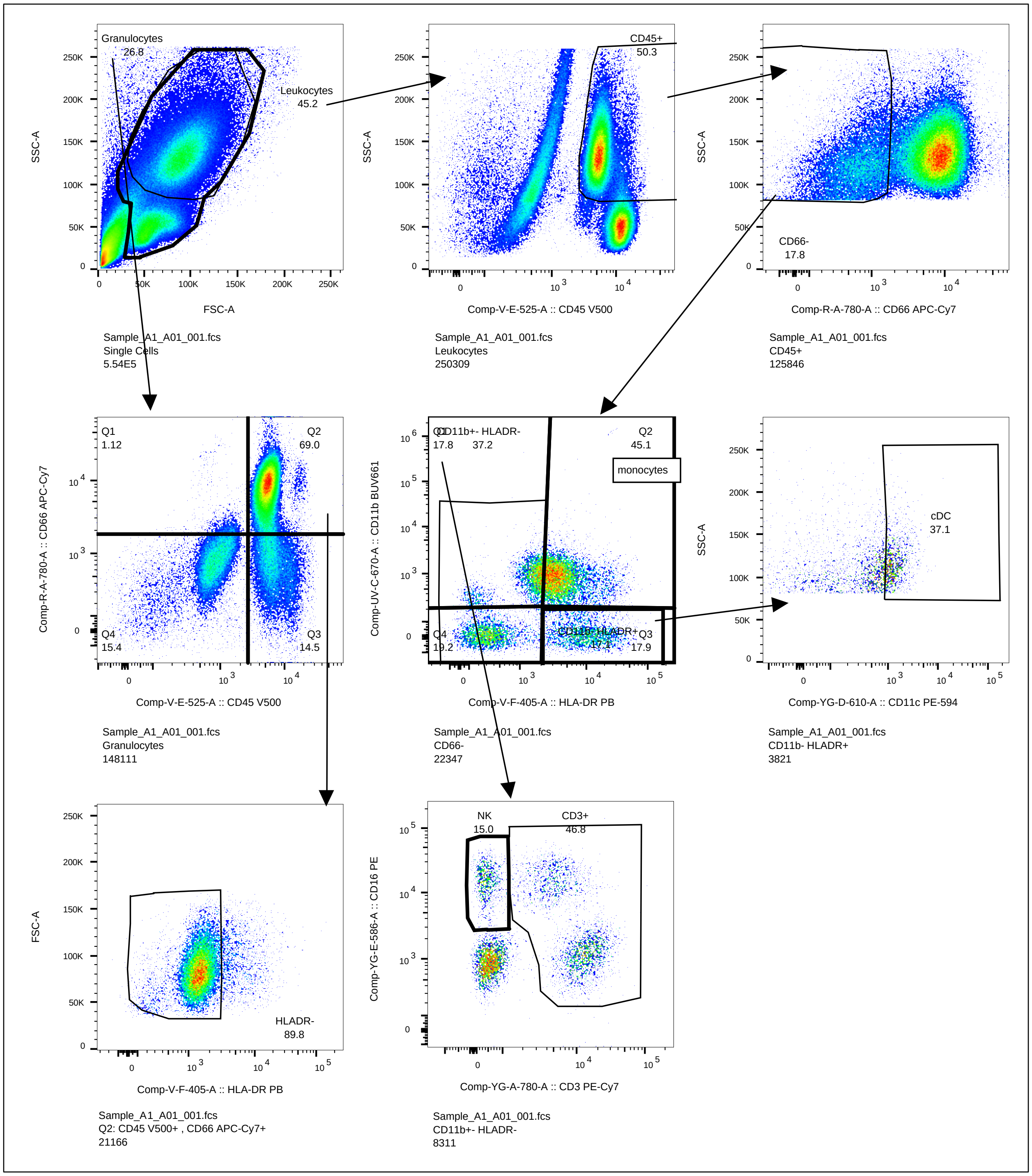

Figure S1. Gating strategy for identifying immune cell subsets. After doublets exclusion and live-dead selection, we used size and granularity scatters (SSC-A, FSC-A) to identify leukocytes and granulocytes. From granulocytes, we further gated for neutrophils based on CD45+ and CD66+ expression. From the leukocytes gate, we gated for CD45+, then excluded CD66. From this CD66- gate, we created quadrant based on HLA-DR and CD11b expression. CD11b+/- and HLA-DR- cells were further gated with CD3 and CD16, separating NK and T lymphocytes. CD11b+ and HLA-DR+ represented monocytes. CD11b- and HLA-DR+ cells were further gated based on CD11c to separate classical DC.

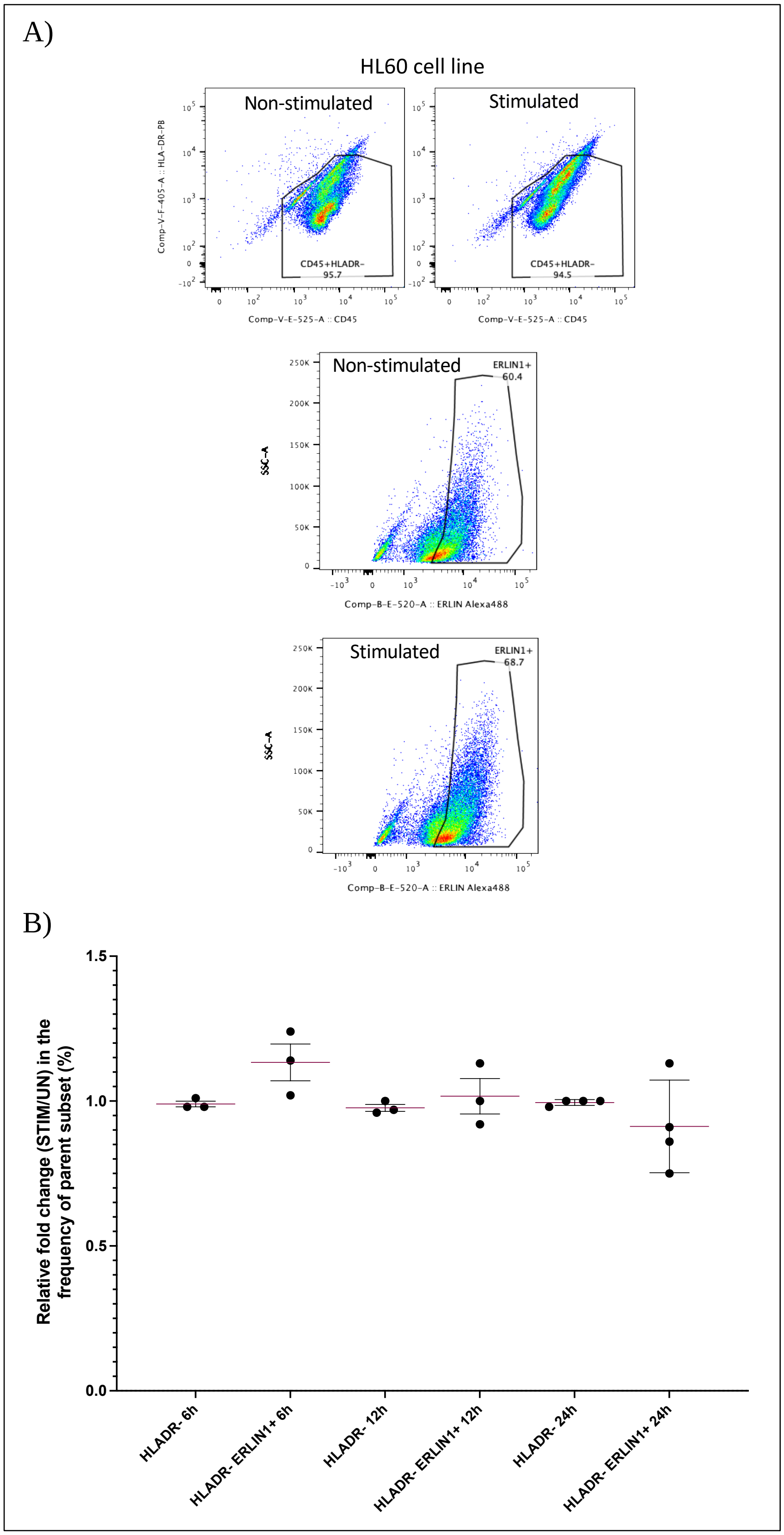

Figure S2. Summary of the temporal expression of ERLIN1 in HL60 cells with and without stimulation assessed by flow cytometry. **A)** Representative expression of ERLIN1 in cultured

HL60 cells before and after stimulation for 24h (expressed as % of parent cell population). Briefly, cells were differentiated with 1.3% DMSO for six days prior to *in vitro* stimulation. After culture, cells were harvested in BD FACSLyse and store at -80°C until staining. Immunostaining and Flow cytometry was performed as described in Methods. **B)** The summary of the main findings shows the relative fold change (STIM/UN) of the percent frequency of the parent subset for each time point.

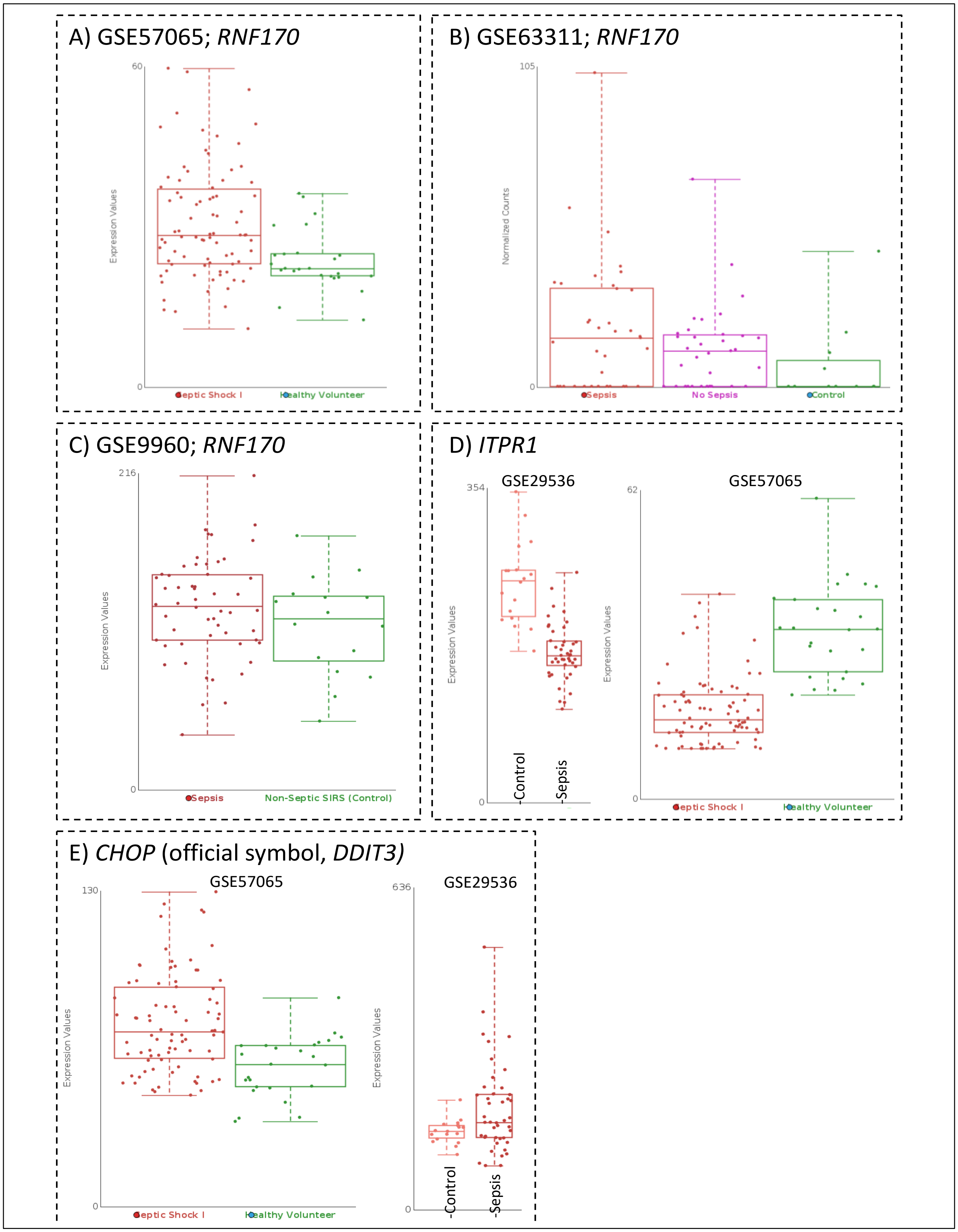

Figure S3. Gene expression data from the indicated GSE ID can be easily visualized on the GXB platform we previously developed as part of our curated transcriptomic dataset collection (unpublished and currently under review, Toufiq *et al.* 2021, SysInflam HuDB).

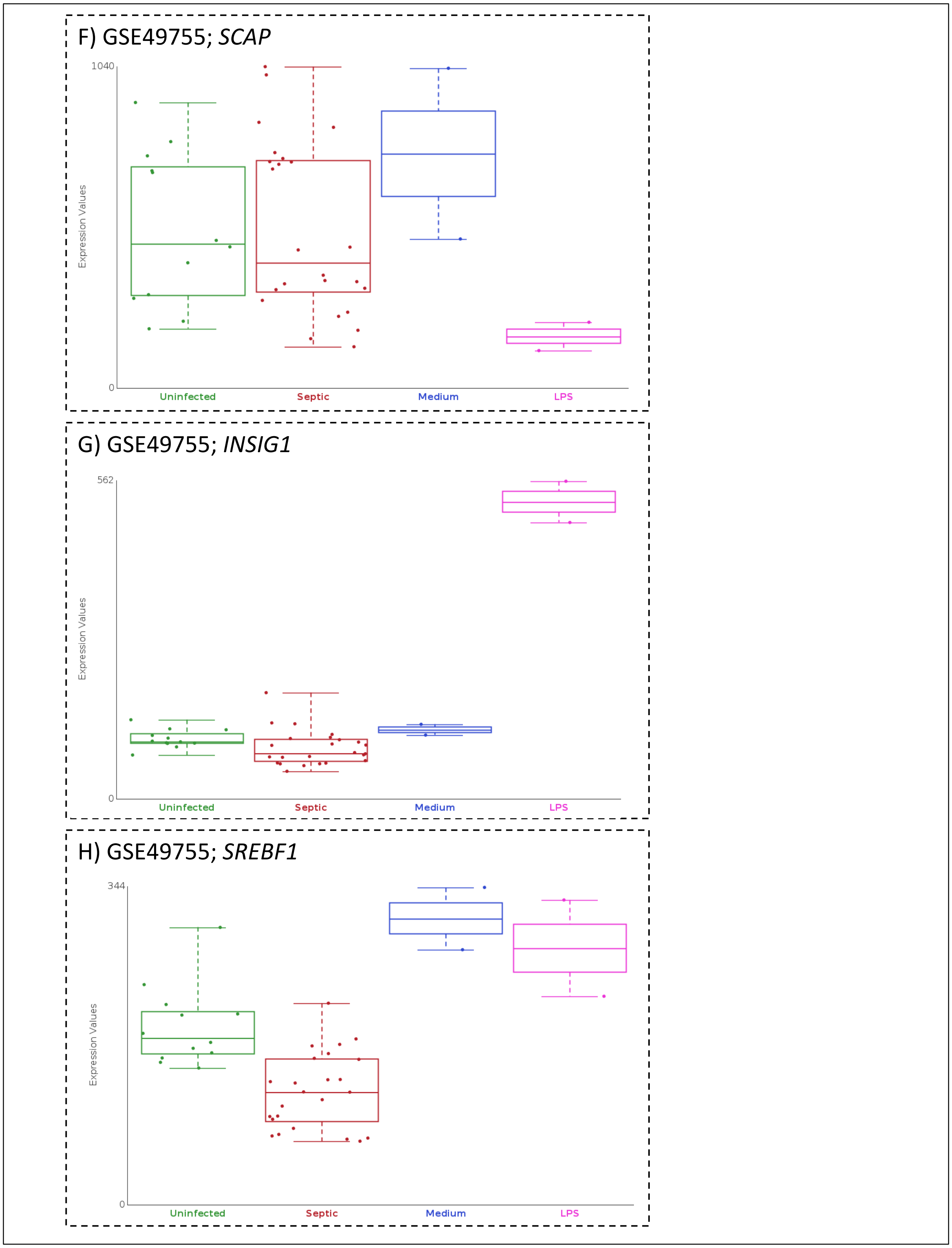

Figure S3. Continued.

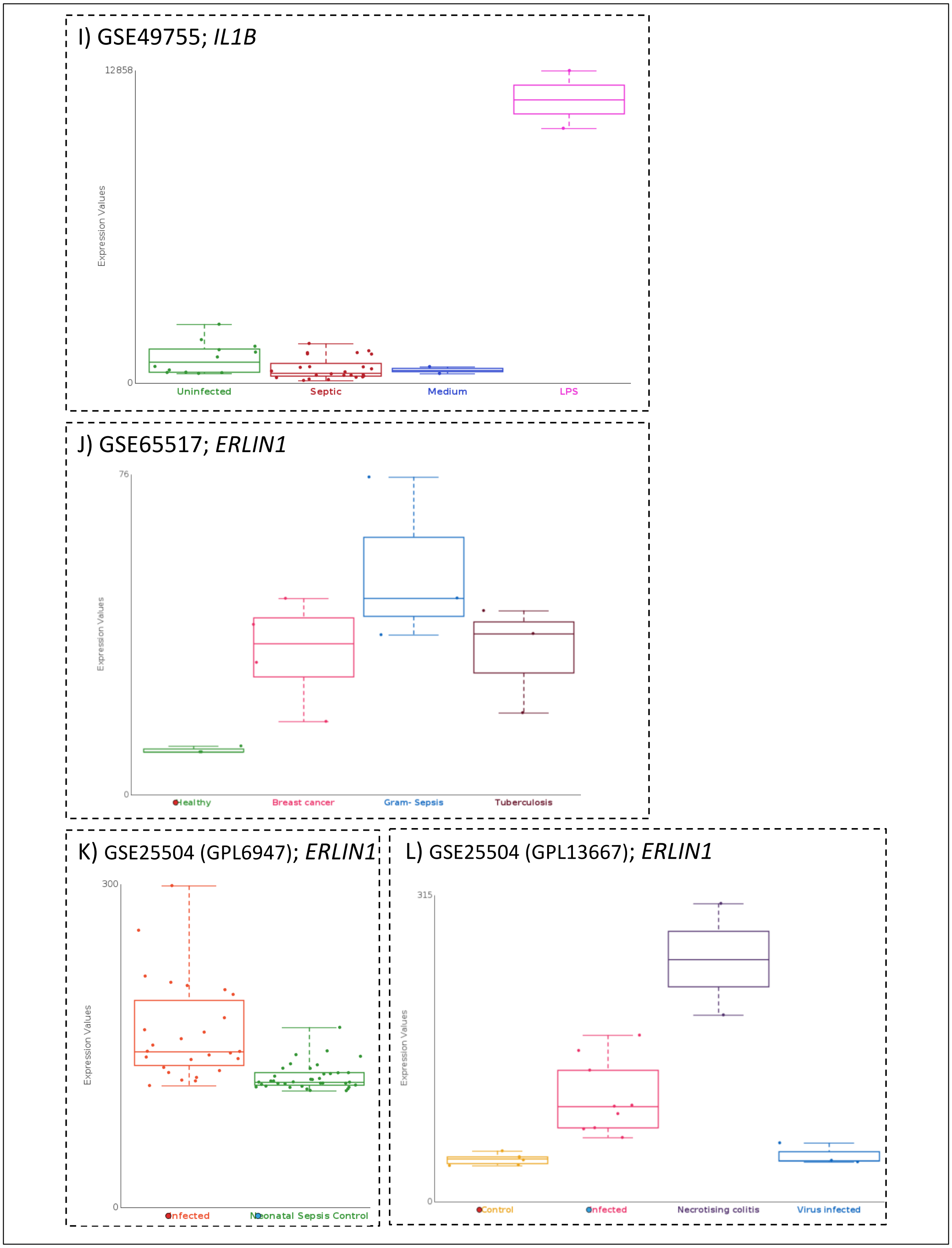

Figure S3. Continued.

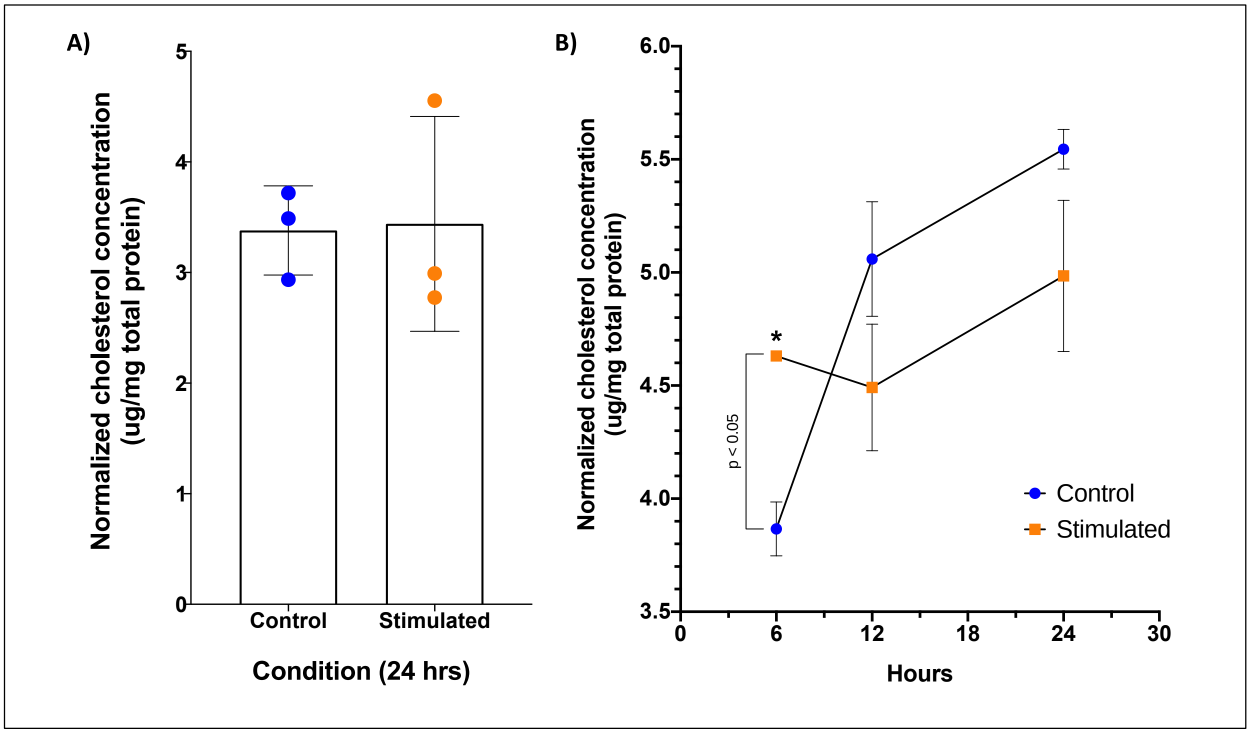

Figure S4. Intracellular cholesterol concentration in cultured whole blood and HL60 cell line exposed to media (control) or a combination of LPS and PGS (see Methods). Total intracellular cholesterol content was quantified using Amplex Red Cholesterol Assay. Each point represents the average of three technical replicates from a blood volunteer (A) or three independent experiments with HL60 cells (B). Shown is the average and standard deviation.

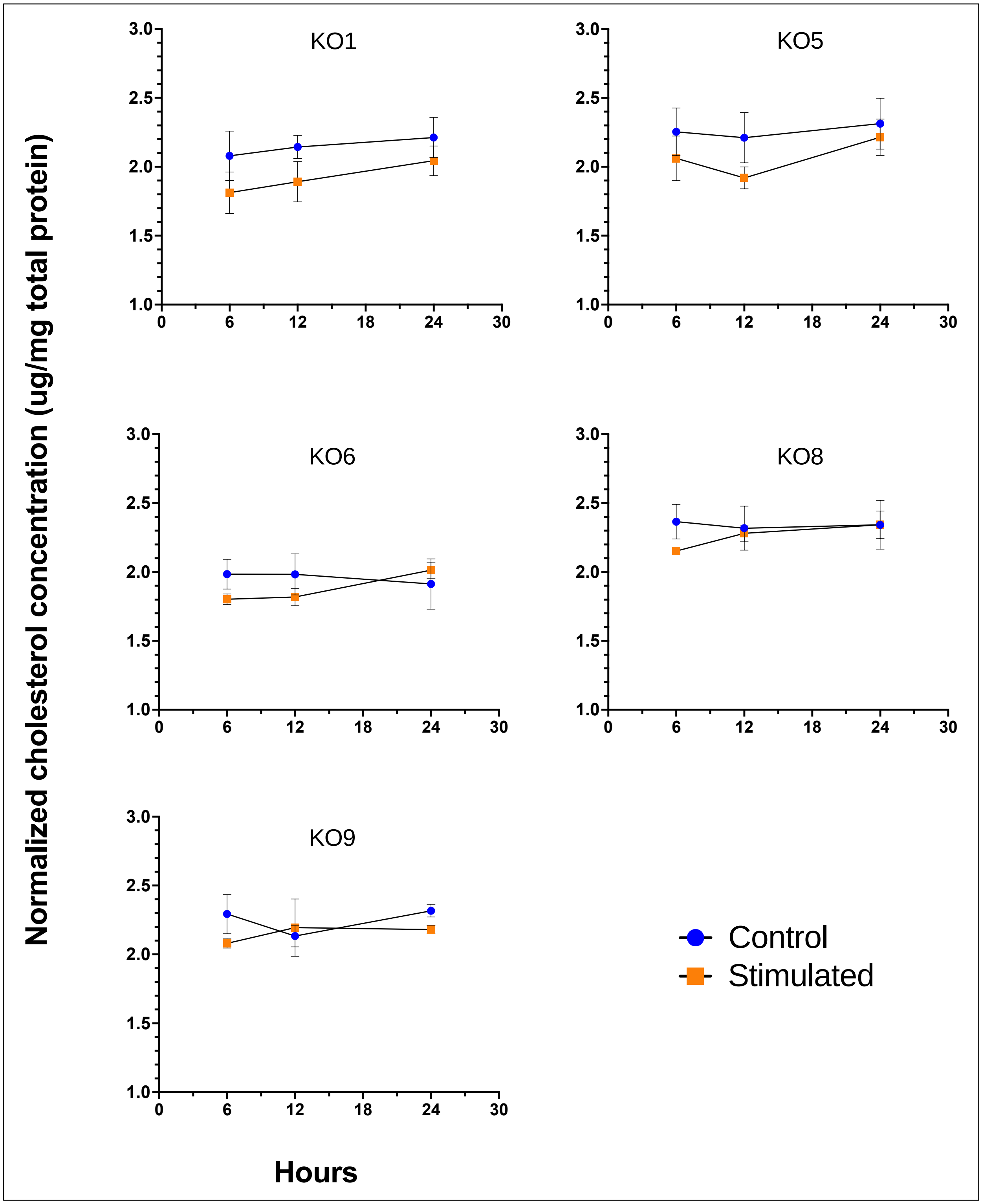

Figure S5. Intracellular cholesterol concentrations in ERLIN1 knock-out HL60 cell lines exposed to media (control) or a combination of LPS and PGS (see Methods). Total intracellular cholesterol content was quantified using Amplex Red Cholesterol Assay. Each point represents the average of three independent experiments with the HL60 knockout cell lines (the knockout clone ID number is indicated on each plot). Shown is the average and standard deviation.
